# Investigating the Skin Health Benefits of *Rosa roxburghii*, *Punica granatum* and Rose: A Randomized Single‐Blind Controlled Clinical Trial

**DOI:** 10.1002/fsn3.4579

**Published:** 2024-10-26

**Authors:** Yining Hao, Zhan Wang, Liping Qu

**Affiliations:** ^1^ Yunnan Botanee Bio‐Technology Group Co., Ltd. Kunming China; ^2^ Shanghai Jiyan Bio‐Pharmaceutical Co., Ltd. Shanghai China; ^3^ FBU Cosmetic Dermatology Clinic Co., Ltd. Shenzhen China; ^4^ Yunnan Yunke Characteristic Plant Extraction Laboratory co., Ltd. Kunming China; ^5^ Medaesthee (Shanghai) Biotechnology co., Ltd. Shanghai China

**Keywords:** *Punica granatum*, *Rosa roxburghii*, rose, skin health

## Abstract

Recent studies underscore the beneficial impacts of oral natural plant extracts on human skin health, though clinical evidence of their efficacy and safety is limited. This study evaluates the skin health effects of a novel oral supplement containing *Rosa roxburghii*, *Punica granatum*, and rose extracts (RPR) 0.70 healthy female participants were randomly assigned to either a control group or an RPR group, with the latter ingesting 20 mL of the RPR supplement daily on an empty stomach over 8 weeks. After 8 weeks, the RPR group exhibited significant enhancements (*p* < 0.001) in skin hydration, glossiness, elasticity, and skin tone, with increases of 69.02%, 30.48%, 25.97%, and 7.52%, respectively. Concurrently, decreases in skin firmness and melanin levels were observed at 21.17% (*p* = 0.007) and 25.06% (*p* < 0.001), respectively. Statistical analysis confirmed that these changes were significantly greater than those in the control group. Image analysis indicated no significant changes in mean optical density of hyperpigmented spots within the RPR group (*p* = 0.367), but a significant reduction in the areas of hyperpigmented spots, under‐eye fine lines, and crow's feet by 41.50%, 37.55%, and 29.36%, respectively (*p* < 0.001), whereas no significant changes were detected in the control group. Importantly, no adverse effects were observed. These findings suggest that the combined intake of *Rosa roxburghii*, *Punica granatum*, and rose extracts can improve skin health, offering a promising natural alternative for dermatological care.

## Graphical abstract

The results demonstrated that the intake of natural plant extracts impacts skin health significantly, as evidenced by improvements in skin hydration, glossiness, elasticity, and reductions in melanin, hyperpigmentation, and wrinkles of participants.

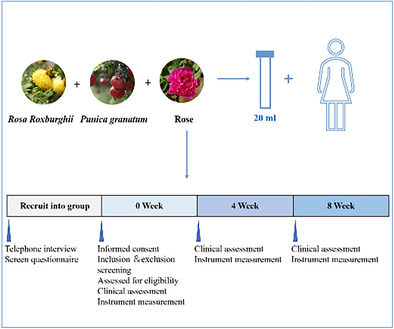

## 1 Introduction

Skin aging, a multifaceted process driven by internal and external factors, manifests through decreased skin elasticity, roughness, wrinkles, dullness, and pigmentation (Tobin 2017). Amidst the burgeoning beauty and nutrition industry, propelled by scientific advancements and the rising “beauty economy,” the efficacy of dietary supplements such as collagen peptides, hyaluronic acid, and Coenzyme Q10 in mitigating skin aging has been recognized (Czajka et al. 2018; Oe et al. 2017; Vollmer, West, and Lephart 2018). Concurrently, the exploration of natural plant extracts as potential agents for skin health enhancement represents a burgeoning field within nutritional science (Fam et al. 2020). Rich in bioactive compounds—vitamins C and E, β‐carotene, polyphenols, and phenolic acids—these plant extracts offer antioxidative benefits, inflammation reduction, and structural support to the skin (Fam et al. 2022).

*Punica granatum*, known for its high concentration of polyphenols, flavonoids, ellagitannins, and punicalagins, has been extensively utilized across functional foods, pharmaceuticals, and skincare for its notable antioxidant and anti‐inflammatory properties (Viuda‐Martos, Fernández‐López, and Pérez‐Álvarez 2010), alongside its ability to shield the skin from ultraviolet (UV) radiation damage (Afaq et al. 2009; Pacheco‐Palencia et al. 2008). *Rosa Roxburghii*, celebrated as a rich source of vitamin C, superoxide dismutase (SOD), polyphenols, and polysaccharides, and thus termed the “king of vitamin C,” (Wang et al. 2021) demonstrates profound antioxidative strength and potential in skin whitening through tyrosinase activity inhibition (Li et al. 2022; Wang et al. 2022). Rose, endowed with polyphenols, flavonoids, and vitamin C, exhibits pharmacological actions that alleviate depression, harmonize blood, and alleviate pain (Pal et al. 2018), with emerging studies highlighting its efficacy in melanin reduction and anti‐aging through the suppression of matrix metalloproteinase‐1 (MMP‐1) levels (Song et al. 2020).

In the context of increasing demands for efficacious, safe, and enduring anti‐aging strategies, leveraging dietary means to prevent and ameliorate skin aging has become a discernible trend. Despite the reported effectiveness of oral nutritional supplements in skin condition improvement, clinical investigations into the application of natural plant extracts as dietary supplements for skin aging prevention and mitigation remain limited. This study seeks to assess the impact of a supplement blend containing *Rosa roxburghii*, *Punica granatum*, and rose extracts on skin health, thereby contributing a theoretical foundation for the diversified exploitation of medicinal plant resources in addressing skin aging.

## 2 Materials and Methods

### 2.1 Study Design

This was a single‐center, randomized, single‐blind, controlled clinical trial over a duration of 8 weeks. The study group consisted of 70 healthy Chinese female participants, aged between 25 and 55, who self‐reported facial skin concerns including dryness, dullness, and reduced elasticity. Subjects were randomly assigned into two groups: a test group, receiving the RPR supplement, and a control group, each comprising 35 individuals with balanced age distribution. The primary objective of this study was to assess the dermatological alterations following 4 and 8 weeks of RPR supplement intake. During the test period, subjects in the RPR group were administered a daily morning beverage on an empty stomach, containing extracts of *Rosa roxburghii* (100 mg), *Punica granatum* (10 mL), and rose (100 mg). Each 20 mL dose of this specially formulated supplement provided 25.00 mg of total polyphenols, 356.40 μg of ellagic acid, and 3.22 mg of vitamin C. Conversely, the control group was not administered any supplement throughout the 8‐week period. To ensure uniformity in skincare routines, which could potentially affect the study's outcomes, all subjects were provided with and instructed to use standardized skincare products contained only moisturizing ingredients and sunscreen, courtesy of the Yunnan Plant Biotechnology Group Co., Ltd. This methodology was meticulously crafted to discern the effects of the RPR supplementation on skin health, controlling for external variables such as skincare product differences.

### 2.2 Ethics Statement

The study was carried out following the rules of the Declaration of Helsinki and approved by the Ethics Committee of Shanghai Clinic Research (Shanghai, China) (approval code: SECCR/2022–091‐01) and Clinical Trials.gov (https://www.clinicaltrials.gov): NCT06274450.

### 2.3 Sample Size

Guided by the central limit theorem, which states that larger sample sizes generally approximate a normal distribution, our study determined a minimum sample size of 30 participants per group based on empirical evidence that sample means tend toward normality when *N* ≥ 30. To accommodate potential losses such as a 15% attrition rate, adherence to exclusion criteria, and to ensure rigorous safety monitoring, the study was designed with a total of 70 participants, allocating 35 individuals to each group.

### 2.4 Participants

Inclusion criteria: (1) Age between 25 and 45 years, healthy Chinese females; (2) reported facial issues such as dryness, roughness, dullness, and lack of elasticity; (3) Body Mass Index (BMI) ranging from 18 to 24 kg/m^2^; (4) clinical assessment by a physician identifies at least one facial hyperpigmented spot with an Individual Typology Angle (ITA°) difference greater than 10° compared to the surrounding skin, with a minimum diameter of 3 mm; (5) facial skin hydration levels, measured by corneometer, ranging from 15 to 45; (6) overall healthy condition with no chronic diseases or ongoing treatments other than skin conditions; (7) willingness to participate in the study, with informed consent provided. Exclusion criteria: (1) Use of any products, dietary supplements, or medications affecting skin coloration within the past 2 months; (2) application of retinoids, chemical peeling, laser treatments, or intense pulsed light therapy at the test site within the last 3 months; (3) unavoidable prolonged exposure to sunlight; (4) history of alcohol abuse or known allergies; (5) participation in any clinical trial within the past month; (6) application of anti‐inflammatory medications on the test site within the past 2 months; (7) history of dermatological conditions such as psoriasis, eczema, scleroderma, or skin cancer; (8) current treatment for asthma or other chronic respiratory diseases; (9) administration of antihistamines either orally or via injection in the past month; (10) severe internal medicine conditions, other significant health issues, or chronic diseases.

### 2.5 Randomization and Blinding

Following eligibility assessment, a data statistician assigned each participant a random number ranging from 1 to 2 using the RANDBETWEEN function in Excel. Participants were then randomly allocated to either Group 1 (RPR group) or Group 2 (control group). The group codes representing the RPR or control group were concealed from the researchers responsible for implementing the study, ensuring that they were unaware of each participant's group assignment.

### 2.6 Evaluation of Skin Conditions

#### 2.6.1 Objective Assessments of Skin Conditions

All participants underwent evaluations before, as well as 4‐ and 8‐week post‐intervention. Prior to each assessment, participants' faces were cleansed and they were allowed to acclimate for 30 min in a controlled environment, maintained at a constant temperature of 21°C ± 1°C and relative humidity of 50% ± 10%. Skin hydration and glossiness levels were quantitatively assessed using a corneometer (Courage & Khazaka, Germany) and a glossymeter (Delfin, Finland), respectively. Skin elasticity and firmness were measured with a Cutometer MPA580 (Courage & Khazaka, Germany), while skin tone (ITA°) and melanin content were determined using a colorimeter and a mexameter MX18 (Courage & Khazaka, Germany), respectively.

#### 2.6.2 Facial Imaging

Facial images of participants were captured using a VISIA‐CR system (CANFIELD, America) under standardized lighting conditions. Analysis was conducted using Image‐Pro Plus 7.0 software. The parameters assessed included the area and proportion of crow's feet, the area and proportion of fine lines under the eyes, the average optical density of hyperpigmented spots, and the proportional area of these spots.

#### 2.6.3 Physician Clinical Evaluation

A clinician scored the density of facial cheek hyperpigmentation (0–7), the severity of cheek hyperpigmentation (0–5), and the isolated facial hyperpigmentation in terms of color difference from surrounding skin and size (0–7), based on a scale in which higher scores indicated greater severity.

### 2.7 Safety

Data on adverse events were collected from the moment participants signed the informed consent form until 14 days post‐study. This included documenting the type, incidence, severity, and relationship to the intervention. Adverse events and serious adverse events were reported by the investigator in an adverse event table included in the case report form.

### 2.8 Statistical Analysis

A t‐test was employed to compare data between groups. For data not conforming to normal distribution, the Wilcoxon signed‐rank test was utilized. All statistical tests were two‐sided, with *p* values calculated accordingly. A *p* value of 0.05 or less was considered to indicate statistical significance. Statistical analyses were conducted using SPSS version 22, and results are presented as mean ± standard error (SE).

## 3 Results

### 3.1 Sample Size Statistics

**FIGURE 1:**
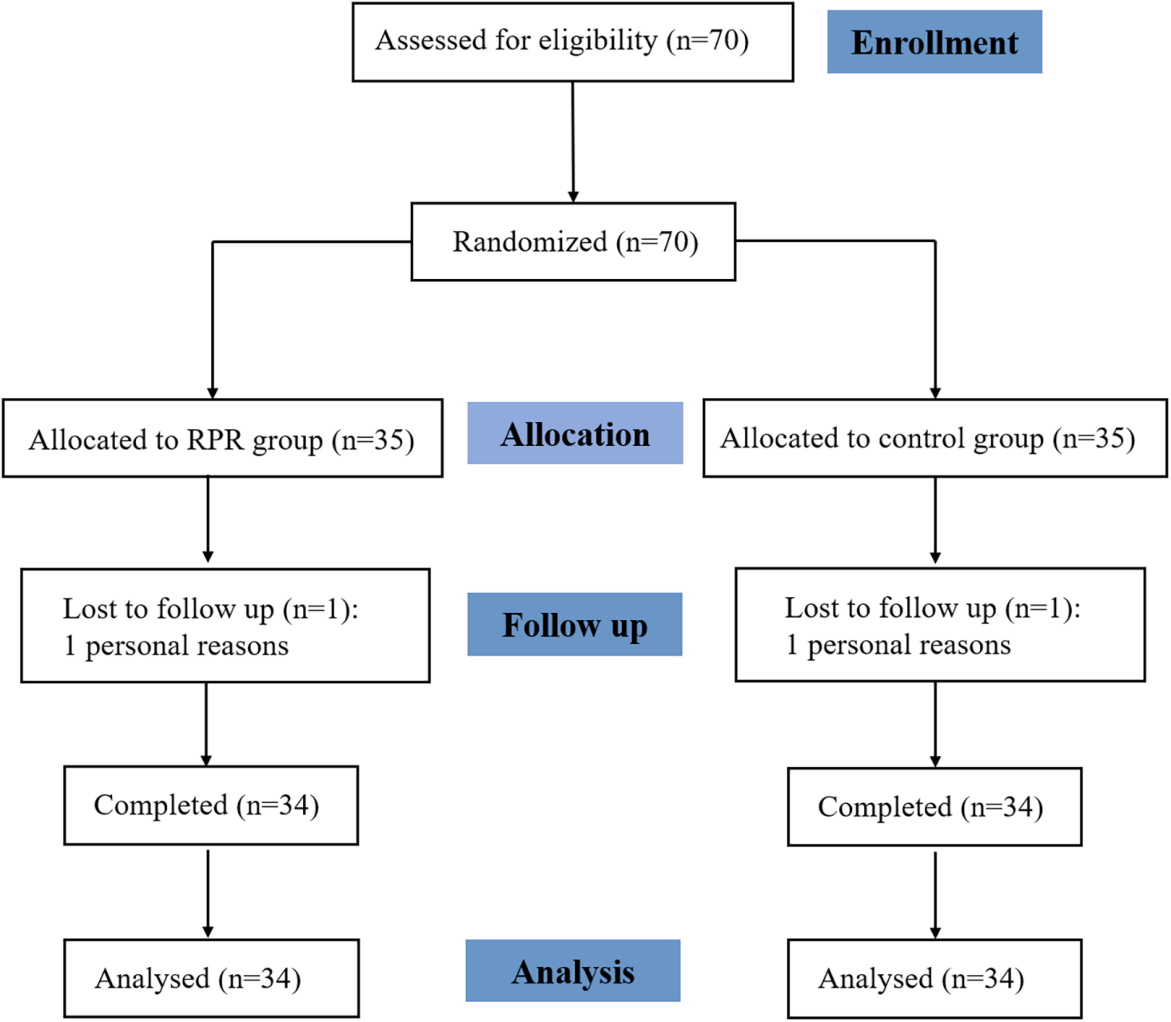
Flowchart depicting the design of the study.

A total of 70 samples met the eligibility criteria, with 68 samples ultimately analyzed as depicted in Figure 1. Statistical analysis revealed the mean age of the study population to be 39.69 ± 4.07 years, with the test group averaging 40.00 ± 4.08 years and the control group 39.38 ± 4.10 years.

### 3.2 Evaluation of Skin Conditions

#### 3.2.1 Skin Hydration and Glossiness

As shown in Table 1, compared to baseline values, skin hydration in the RPR group exhibited significant increases of 38.53% (*p* < 0.001) after 4 weeks and 69.02% (*p* < 0.001) after 8 weeks (Table 2). Similarly, skin glossiness increased by 16.24% after 4 weeks and 30.48% after 8 weeks (*p* < 0.001). Statistically significant differences were observed in the changes in skin hydration and glossiness between the RPR group and the control group at both 4 and 8 weeks (*p <* 0.001).

**TABLE 1:** Effects of RPR supplementation on skin hydration, radiance, elasticity, firmness, tone, roughness, and melanin.

Items	Time (week)	RPR group (*n* = 34)			Control group (*n* = 30)			*p* value for change scores (PZG group vs. placebo group)
		Mean ± SE	*p* value (vs. baseline)	Change (vs. baseline)	Mean ± SE	*p* value (vs. baseline)	Change (vs. baseline)	
Skin hydration	0	32.36 ± 0.93			34.02 ± 0.71			
	4	44.83 ± 0.87	0.000	12.47 ± 0.70	42.47 ± 0.59	0.006	8.45 ± 0.57	0.000
	8	54.70 ± 1.17	0.000	22.34 ± 0.73	52.15 ± 0.82	0.000	18.13 ± 0.60	0.000
Skin radiance	0	5.69 ± 0.12			5.63 ± 0.14			
	4	6.61 ± 0.13	0.000	0.92 ± 0.06	6.22 ± 0.14	0.000	0.59 ± 0.09	0.002
	8	7.42 ± 0.13	0.000	1.73 ± 0.08	7.05 ± 0.13	0.000	1.42 ± 0.10	0.020
Skin elasticity R2	0	0.59 ± 0.01			0.61 ± 0.02			
	4	0.60 ± 0.01	0.390	0.02 ± 0.02	0.60 ± 0.02	0.563	−0.01 ± 0.02	0.306
	8	0.74 ± 0.01	0.000	0.15 ± 0.02	0.68 ± 0.02	0.001	0.07 ± 0.02	0.001
Skin firmness F4	0	7.18 ± 0.31			7.01 ± 0.23			
	4	5.93 ± 0.25	0.003	−1.25 ± 0.39	6.47 ± 0.24	0.046	−0.53 ± 0.26	0.133
	8	5.66 ± 0.19	0.000	−1.52 ± 0.36	6.46 ± 0.16	0.007	−0.55 ± 0.19	0.020
Skin tone ITA°	0	46.28 ± 1.17			46.73 ± 1.29			
	4	45.89 ± 1.05	0.552	−0.39 ± 0.64	45.20 ± 1.43	0.066	−1.53 ± 0.84	0.285
	8	49.76 ± 1.17	0.000	3.48 ± 0.81	47.21 ± 1.42	0.596	0.48 ± 0.93	0.018
Skin melanin	0	209.51 ± 5.04			205.31 ± 7.28			
	4	180.97 ± 5.53	0.000	−28.54 ± 3.64	195.43 ± 7.72	0.002	−9.88 ± 3.99	0.000
	8	157.01 ± 4.50	0.000	−52.50 ± 4.38	190.67 ± 8.39	0.001	−14.65 ± 4.54	0.000

**TABLE 2:** Effects of RPR supplementation on skin hyperpigmented spots, fine lines under the eyes and crow's feet.

Items	Time (week)	RPR group (*n* = 34)			Control group (*n* = 30)			*p* value for change scores (PZG group vs. placebo group)
		Mean ± SE	*p* value (vs. baseline)	Change (vs. baseline)	Mean ± SE	*p* value (vs. baseline)	Change (vs. baseline)	
Mean optical density of hyperpigmented spots	0	94.04 ± 1.98			93.17 ± 1.78			
	4	94.22 ± 1.36	0.270	0.18 ± 1.34	93.24 ± 1.99	0.905	0.08 ± 0.59	0.286
	8	94.44 ± 1.72	0.367	0.40 ± 1.69	94.66 ± 1.95	0.064	1.50 ± 0.75	0.783
Proportional area of hyperpigmented spots (%)	0	21.15 ± 1.85			17.24 ± 1.87			
	4	15.73 ± 1.42	0.000	−5.42 ± 0.89	16.36 ± 1.70	0.330	−0.88 ± 0.70	0.000
	8	12.37 ± 1.30	0.000	−8.78 ± 0.97	16.25 ± 1.69	0.966	−0.99 ± 1.08	0.000
Proportional area of fine lines under the eyes (%)	0	14.93 ± 1.16			12.79 ± 0.92			
	4	12.73 ± 0.96	0.000	−2.19 ± 0.48	11.62 ± 0.85	0.014	−1.17 ± 0.45	0.123
	8	9.32 ± 0.80	0.000	−5.61 ± 0.59	11.23 ± 0.85	0.016	−1.56 ± 0.62	0.000
Proportional area of crow's feet (%)	0	12.95 ± 0.59			11.09 ± 0.72			
	4	11.10 ± 0.53	0.000	−1.85 ± 0.26	10.82 ± 0.64	0.381	−0.27 ± 0.31	0.000
	8	9.15 ± 0.54	0.000	−3.80 ± 0.37	10.69 ± 0.64	0.326	−0.40 ± 0.40	0.000

#### 3.2.2 Skin Elasticity and Firmness

In this study, skin elasticity and firmness were quantified using a Cutometer@MpA580. The probe evaluated overall elasticity (R2) and firmness (F4) parameters, where a value closer to 1 for R2 suggests better elasticity, and a lower value for F4 indicates superior resistance to suction, reflecting increased firmness. The results indicated that in the RPR group, the overall skin elasticity significantly improved by 25.97% after 8 weeks (*p* < 0.001), while changes at 4 weeks were not statistically significant (*p* = 0.563) (Table 1). Furthermore, the improvement in R2 at 8 weeks was significantly greater in the RPR group compared to the control group (*p* ≤ 0.001). Regarding skin firmness decreased by 17.37% at 4 weeks (*p* = 0.046) and further declined by 21.17% at 8 weeks (*p* = 0.007). Furthermore, the reduction in skin firmness at 8 weeks in the experimental group was found to be significantly different from that in the control group (*p* ≤ 0.05).

#### 3.2.3 Skin Tone and Melanin

Increases in the ITA° value indicate enhanced skin brightness. As shown in Table 1, compared to baseline values, the ITA° in the RPR group significantly increased by 7.52% after 8 weeks (*p* < 0.001), with no significant change observed after 4 weeks (*p* = 0.552). In contrast, the control group did not show significant changes in ITA° either after 4 weeks (*p* = 0.066) or 8 weeks (*p* = 0.596). The change in ITA° at 8 weeks in the RPR group was significantly greater than that in the control group (*p* = 0.018). Moreover, compared to baseline, melanin levels in the RPR group decreased significantly by 13.62% after 4 weeks (*p* < 0.001) and by 25.06% after 8 weeks (*p* < 0.001), with changes notably exceeding those in the control group (*p* < 0.001).

#### 3.2.4 Skin Pigment Spots

**FIGURE 2:**
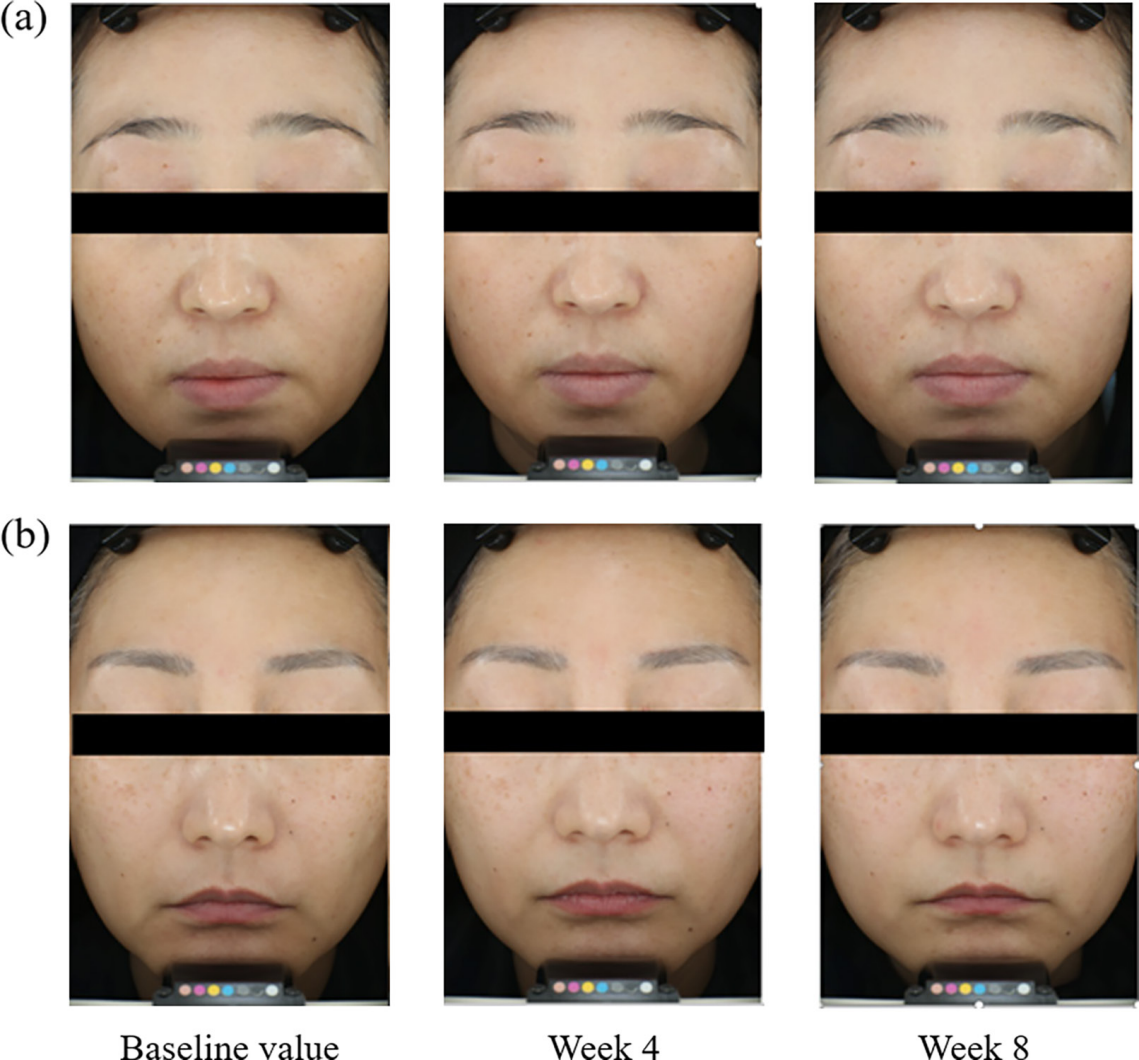
Facial images of test group at baseline and 4 and 8 weeks, respectively. (a) Subject number: 048, age: 43 years; (b) subject number: 048, age: 43 years.

Image analysis results, as depicted in Figure 2 and summarized in Table 2, revealed that there were no significant changes in the average optical density of hyperpigmented spots in both the RPR and control groups after 4 and 8 weeks compared to baseline values (*p* > 0.05). However, a significant reduction was observed in the proportional area of these spots. In the RPR group, the area decreased by 25.61% after 4 weeks (*p* < 0.001) and by 41.50% after 8 weeks (*p* < 0.001). No significant changes in the area of hyperpigmented spots were observed in the control group after 4 weeks and 8 weeks (*p* > 0.05).

#### 3.2.5 Skin Crow's Feet and Fine Lines

In the RPR group, the proportional area of crow's feet significantly decreased compared to baseline (*p* < 0.001), with reductions of 14.27% after 4 weeks and 29.36% after 8 weeks (Table 2). In contrast, the control group showed no significant changes in the area of crow's feet after both 4 and 8 weeks (*p* > 0.05). Additionally, the area proportion of under‐eye fine lines in the RPR group significantly decreased by 37.55% after 8 weeks compared to baseline (*p* < 0.001). The change in the area of under‐eye fine lines after 8 weeks was also significantly greater in the experimental group than in the control group (*p* < 0.001).

#### 3.2.6 Clinical Assessment of Hyperpigmentation

In the RPR group, significant reductions were observed in the clinical assessment scores for cheek hyperpigmentation density, severity of cheek hyperpigmentation, and the color difference between isolated facial hyperpigmentation and surrounding skin after both 4 and 8 weeks compared to baseline (*p* ≤ 0.05) (Table 3). In contrast, the control group showed no significant changes in these parameters after 4 and 8 weeks (*p* > 0.05). No significant differences were found in the changes in cheek hyperpigmentation density, severity, or isolated facial hyperpigmentation color differences between the RPR group and the control group after 4 and 8 weeks (*p* > 0.05). However, the size of isolated facial hyperpigmentation in the RPR group significantly decreased after 8 weeks compared to baseline (*p* = 0.014), with a change significantly greater than that observed in the control group (*p* = 0.048).

**TABLE 3:** Effects of RPR supplementation on hyperpigmentation density, severity of hyperpigmentation, color contrast between pigmented lesion and adjacent skin, and size of pigmented spot.

Items	Time (week)	RPR group (*n* = 34)			Control group (*n* = 30)			*p* value for change scores (PZG group vs. placebo group)
		Mean ± SE	*p* value (vs. baseline)	Change (vs. baseline)	Mean ± SE	*p* value (vs. baseline)	Change (vs. baseline)	
Hyperpigmentation density	0	2.21 ± 0.13			2.47 ± 0.21			
	4	2.03 ± 0.14	0.014	−0.18 ± 0.07	2.44 ± 0.21	0.564	−0.03 ± 0.05	0.087
	8	2.03 ± 0.15	0.014	−0.18 ± 0.07	2.38 ± 0.20	0.083	−0.09 ± 0.05	0.287
Severity of hyperpigmentation	0	2.18 ± 0.09			2.38 ± 0.14			
	4	2.03 ± 0.11	0.025	−0.15 ± 0.06	2.41 ± 0.15	0.655	−0.88 ± 0.70	0.060
	8	2.03 ± 0.11	0.025	−0.15 ± 0.06	2.41 ± 0.16	0.655	−0.99 ± 1.08	0.060
Color contrast between pigmented lesion and adjacent skin	0	2.06 ± 0.14			2.09 ± 0.14			
	4	1.91 ± 0.12	0.025	−0.15 ± 0.06	2.00 ± 0.14	0.083	−0.09 ± 0.05	0.455
	8	1.82 ± 0.12	0.011	−0.24 ± 0.09	2.00 ± 0.14	0.083	−0.09 ± 0.05	0.165
Size of hyperpigmented Spots	0	2.12 ± 0.13			2.32 ± 0.17			
	4	2.06 ± 0.14	0.157	−0.06 ± 0.04	2.24 ± 0.17	0.083	−0.09 ± 0.05	0.645
	8	1.94 ± 0.13	0.014	−0.18 ± 0.07	2.29 ± 0.17	0.317	−0.09 ± 0.05	0.048

### 3.3 Adverse Events/Serious Adverse Event Records

No adverse events or serious adverse events were reported during the study or within the 56‐day follow‐up period after the study's conclusion.

## 4 Discussion

Our study demonstrated significant improvements in skin hydration and glossiness after 4 and 8 weeks of consuming the RPR supplement (*p* < 0.001). These results align with previous research where the intake of beverages containing lingonberry and blueberry extracts led to significant hydration increases in 33 healthy women over 12 weeks, likely due to increases in hyaluronic acid and natural moisturizing factors (NMFs) (Uchiyama et al. 2019). Additionally, other studies indicate that oral supplements like collagen, ceramides, hyaluronic acid, and proanthocyanins can also enhance skin hydration (Sun et al. 2022). As the body's largest organ, the skin plays a critical role in protection, thermoregulation, and immune response (Dąbrowska et al. 2018). However, with aging, the proliferation of epidermal keratinocytes declines, impairing the skin barrier and leading to thinning, dryness, and reduced perspiration—common signs of aging (Gu et al. 2020). Maintaining adequate water content in the stratum corneum is essential for preserving the structural and functional integrity of the skin (Verdier‐Sévrain and Bonté, 2007).

Skin aging is a complex biological process, categorized into intrinsic and extrinsic aging (Tigges et al. 2014). During this process, the dermis experiences a reduction in fibroblast numbers and a decrease in the synthetic capacity for collagen and elastin, especially Types I and III, resulting in thinner dermis, reduced skin elasticity, and increased wrinkling (Gu et al. 2020).Clinical results from this study indicate that after 8 weeks of continuous consumption of the RPR supplement, there was a significant increase in overall skin elasticity (R2) and a marked reduction in skin firmness (F4) and the area of under‐eye fine lines and crow's feet (*p* < 0.001). These results suggest the natural plant ‘s potential to prevent and delay skin aging. Dietary factors play a crucial role in regulating skin health. Recent studies have indicated that regular consumption of almonds by postmenopausal women significantly reduces facial wrinkles (Foolad et al. 2019). Additionally, aloe vera extract has been reported to enhance skin elasticity and reduce facial wrinkles by suppressing collagen degradation via downregulation of matrix metalloproteinase‐1 (MMP‐1) expression (Cho et al. 2009).

Reactive oxygen species (ROS) are primary contributors to skin aging. UV radiation not only leads to the accumulation of ROS but also promotes the proliferation of melanocytes and stimulates melanogenesis (Wang et al. 2022). After 8 weeks, the RPR group showed a significant improvement in skin color ITA° and marked reductions in melanin levels, and the size and proportion of hyperpigmented spots, with these changes being significantly greater than in the control group. These results suggest that RPR has the potential to prevent melanogenesis and lighten skin tone. The polyphenols in *Rosa roxburghii*, *Punica granatum*, and rose extracts are potent natural antioxidants, can provide protection against UVA and UVB‐induced human skin fibroblast cell death by reducing intracellular ROS and enhancing antioxidant defenses (Viuda‐Martos, Fernández‐López, and Pérez‐Álvarez 2010; Wang et al. 2021; Nowak et al. 2014). Tyrosinase, a key rate‐limiting enzyme in melanogenesis, is effectively inhibited by pomegranate extracts containing ellagic acid, which reduces UV radiation‐induced pigmentation in guinea pig skin, suggesting a potential skin whitening effect of oral pomegranate extract (Yoshimura et al. 2005). Additionally, the vitamin C‐rich *Rosa roxburghii* not only plays a critical role in collagen synthesis but also provides photoprotection (Pullar, Carr, and Vissers 2017; Sanadi and Deshmukh 2020). Continuous intake of vitamin C for 8 weeks has been shown to lighten skin tone in young smokers by inhibiting skin cell aging and enhancing the antioxidant activity of high‐density lipoproteins (Kim et al. 2015).

Clinical assessments observed that the RPR group exhibited significant reductions in the density and severity of cheek hyperpigmentation, as well as in the color contrast between isolated hyperpigmented spots and surrounding skin after an 8‐week treatment period. Nevertheless, these improvements did not statistically differ from those seen in the control group. Image analysis corroborated these clinical findings. These findings suggest that observed effects on skin hyperpigmentation might be attributed to the application of topical skincare products rather than to the ingestion of the RPR supplement.

While the findings of this study contribute valuable insights into the effects of natural plant extracts on skin health, several limitations must be acknowledged. The absence of a placebo control group limits our ability to definitively attribute the observed dermatological changes solely to the RPR intervention, introducing potential biases. Nonetheless, the inclusion of control groups allowed for an evaluative comparison between the effects of oral intake and skincare products. Another limitation is that we did not control for or assess participants' daily physical activity levels and dietary intake. These lifestyle factors can significantly influence skin health and might have affected the outcomes observed in this study. Moreover, the small sample size of 70 female participants limits the generalizability of the results to broader populations, including males, and the relatively short duration of 56 days may not fully capture the long‐term effects and sustainability of the observed benefits. These factors underline the need for larger, longer‐term studies with diverse participant demographics, alongside detailed lifestyle assessments, to robustly validate the findings.

## 5 Conclusions

In conclusion, the intake of natural plant extracts impacts skin health significantly, as evidenced by improvements in skin hydration, glossiness, elasticity, and reductions in melanin, hyperpigmentation, and wrinkles of participants. This study supports the use of natural plant extracts in the development and application of skin health products.

## Author Contributions

**Liping Qu:** conceptualization (equal), funding acquisition (equal), project administration (equal), resources (equal), supervision (equal), writing – review and editing (equal).

## Informed Consent Statement

All participants in the study provided informed consent.

## Conflicts of Interest

The authors declare no conflicts of interest.

## Data Availability

The data will be made available upon request.
